# Environmentally enriched housing conditions affect pig welfare, immune system and gut microbiota in early life

**DOI:** 10.1186/s42523-021-00115-2

**Published:** 2021-07-28

**Authors:** Caifang Wen, Ingrid van Dixhoorn, Dirkjan Schokker, Henri Woelders, Norbert Stockhofe-Zurwieden, Johanna M. J. Rebel, Hauke Smidt

**Affiliations:** 1 Laboratory of Microbiology, Wageningen University & Research, Wageningen, The Netherlands; 2 Wageningen Livestock Research, Wageningen University & Research, Wageningen, The Netherlands; 3 Wageningen Bioveterinary Research, Wageningen University & Research, Lelystad, The Netherlands

**Keywords:** Pigs, Enrichment, Behaviour, Immunity, Gut microbiota, Early life

## Abstract

**Background:**

Conventional pig housing and management conditions are associated with gastrointestinal pathophysiology and disease susceptibility in early life. Developing new strategies to reduce both therapeutic and prophylactic antibiotic use is urgent for the sustainable swine production globally. To this end, housing methodology providing effective environmental enrichment could be a promising alternative approach to reduce antibiotic usage, as it has been proven to positively influence pig welfare and immune status and reduce susceptibility to infections. It is, however, poorly understood how this enriched housing affects systemic and local pulmonary immune status and gut microbiota colonization during early life. In the present study, we compared the effects of two housing conditions, i.e., conventional housing: (CH) versus enriched housing (EH), on immune status and gut microbiota from birth until 61 days of age.

**Results:**

The expected benefits of enrichment on pig welfare were confirmed as EH pigs showed more positive behaviour, less aggression behaviour during the weaning transition and better human animal relation during the post weaning phase. Regarding the pigs’ immune status, EH pigs had higher values of haemoglobin and mean corpuscular volume in haematological profiles and higher percentages of T cells and cytotoxic T cells in peripheral blood. Furthermore, EH pigs showed higher ex vivo secretion of IL1ß and TNF-α after lipopolysaccharide stimulation of whole blood than CH pigs. The structure of the developing faecal microbiota of CH and EH pigs significantly differed as early as day 12 with an increase in the relative abundance of several bacterial groups known to be involved in the production of short chain fatty acids, such as *Prevotella*_2, *Christensenellaceae*_R_7_group and *Ruminococcus gauvreauii* group. Furthermore, the main difference between both housing conditions post weaning was that on day 61, CH pigs had significantly larger inter-individual variation of ileal and colonic microbiota than EH pigs. In addition to housing, other intrinsic factors (e.g., sex) were associated with gut microbiota development and immune competence.

**Conclusions:**

In addition to the known welfare benefits for pigs, environmentally enriched housing also positively drives important aspects of the development of the immune system and the establishment of gut microbiota in early life. Consequently, EH may contribute to increasing productivity of pigs and reducing antibiotic use.

**Supplementary Information:**

The online version contains supplementary material available at 10.1186/s42523-021-00115-2.

## Background

Pigs reared under conventional conditions of intensive production are subjected to various sources of stress, including limitations to express natural behaviour like socialization, exploration and rooting [1, 2]. This psychosocial stress has been considered as an important risk factor driving gastrointestinal pathophysiology and disease susceptibility [3–5]. Recently, animal welfare has become of increasing concern to the swine industry as well as the general public [2], and the urgency for a reduction in the use of antibiotics in livestock has become evident with the development and spread of antimicrobial resistance [6–8]. Therefore, effective environmental enrichments in the farm, such as larger spaces, and the provision of rooting substrates, has been proposed as a potential strategy to improve pig welfare, as well as health. The benefits for animal welfare by providing enrichments have been well established in research [9]. A growing number of studies have demonstrated that enriched housing can also positively influence the level of natural (auto) antibodies [5, 10, 11]. Enriched housing has also been shown to affect specific antibody response and blood leukocyte subpopulations in pigs [12]. However, the effectiveness and mechanisms underlying this strategy are still largely unknown. Questions remain, such as how these differences in immune response and blood leucocyte arise, when pigs are kept under enriched housed conditions during early life.

The gut microbiota has been shown to play a pivotal role in immune development and to have impacts on health in humans and animals [13]. Furthermore, the gut microbiota has emerged as a key player in the regulation of the bidirectional communication network of the gut-brain axis (GBA) [14, 15]. Reviewed by Molina-Torres et al. [16], a growing body of literature from human clinical studies and animal models has demonstrated the influence of stress on gut microbiota, and the role of the gut microbiota in stress modulation for different stressors through the GBA. To this end, the hypothalamic–pituitary–adrenal (HPA) axis seems to be the most important pathway for stress response by releasing glucocorticoid hormones, which can influence the gut microbiota, as well as the immune response. Based on accumulating knowledge from rodent and human studies about the influence of the microbiota-gut-brain axis (MGBA) on behaviour, Kraimi et al. [17] have recently reviewed the role of the MGBA in the welfare of farm animals and suggested it is urgent to improve our understanding as to how the MGBA affects behaviours for farm animals. Animal welfare and health could be improved via genetic selection, nutrition, housing, as well as through stress management that takes the role of the gut microbiota in behaviour into account [17]. In pigs, many studies have shown the adverse effects of conventional housing conditions of intensive farms on behaviour, the reactivity of the HPA axis and welfare [2, 4, 7, 10, 18]. Furthermore, in a previous study it was found that piglets reared under housing conditions with social and environmental enrichment showed lower disease susceptibility to co-infection of porcine reproductive and respiratory syndrome virus (PRRSV) and *Actinobacillus pleuropneumoniae* [3]. This social and environmental enrichment was shown to positively influence systemic and lung immune response, as well as clinical outcomes in pigs. Therefore, in this follow-up study we aimed to address how this enriched housing influences gut microbiota colonization of pigs during early and later life, and how housing type dynamically affects the systemic immune status and local pulmonary immune response. Furthermore, we investigated the possible linkage between gut microbiota composition and systemic immune status and local pulmonary immune response of pigs when kept under enriched or conventional housing conditions.

## Methods

### Experimental design, animals and housing

For this experiment the offspring (96 male and female piglets) of eight multiparous Topigs N-line x Z-line York sows (range parity: 2–6) that were inseminated with Temp boar (Topigs) was used at the research facility of Wageningen University & Research, Sterksel, the Netherlands. The sows were inseminated on the same day, and the expected parturition day (10th of January 2017) was defined as day 0 for all piglets. The sows were kept at 2.81 m^2^/sow with 41% solid floor. One week before parturition the sows were moved to farrowing pens, in which the sows were housed in farrowing crates. Four sows with their litters were subjected from the first day of life onwards to conventional housing (conventionally housed piglets: CH), while four other litters were exposed to enriched housing (enriched housed piglets: EH). CH piglets were housed according to current legal requirements for farmed pigs in 5 m^2^ conventional pens with 100% slatted floor and a 100 × 45 cm solid rubber floor mat. EH piglets were housed in 10 m^2^ enriched pens with partly slatted (40%) and partly solid (60%) floor. In the conventional pens two chains were added as enrichment. Enrichment in the enriched pens consisted of straw, moist peat, wood shavings, jute bags and branches of a broom, and was provided and replenished as described previously [3]. All enrichment materials were sterilized by γ-irradiation. A heating lamp for the piglets was provided in each pen during the first week after birth. On day 3, the piglets received an ear tag, they were treated with ironject® 20% + B12 (Dopharma, Raamsdonksveer, the Netherlands) and Baycox (Bayer, Animal Health), and tails were shortened according to standard procedures to prevent tail biting. The male piglets were not castrated.

All enriched and conventional pens had two drinking nozzles, one for the sow and one for the piglets. Sows were fed a standard commercial diet twice a day at 8:00 am and 3:15 pm. The piglets received solid food ad libitum, starting at day 3. Lights were on between 7:00 am and 9:00 pm. Temperature was kept at 25 °C during the first week after birth, and it was decreased by 1 °C every week until it reached 22 °C, the week before weaning.

From day 15 until weaning, the panels between two adjacent enriched pens were removed, allowing piglets from two different EH litters to mingle. Thus, the individual enriched pens of 10 m^2^ were temporarily transformed into pens of 20 m^2^ to enable early social interaction between EH litters.

At day 20 all piglets were vaccinated against circovirus and mycoplasma (Ingelvac CircoFLEX and MycoFLEX; Boehringer Ingelheim Vetmedica). On day 28, the piglets were weaned and moved to weaning pens in which they were regrouped within the housing treatment they had been submitted to, thus forming four new groups per treatment, (eight groups) of 12 piglets each. The piglets were equally mixed taking sex and body weight into account to obtain experimental subgroups with comparable composition in which housing treatment remained the same as before weaning. Temperature was increased to 28 °C at the day of weaning and was decreased by 2 °C each week until it reached 22 °C. Temperature was kept at 22 °C from day 42 to day 63.

On day 61 two pigs per pen were euthanized by injecting pentobarbital (Euthasol 40%, AST Farma) in the auricular vein, while they were restrained and thereafter exsanguinated.

### Behavioural observations, human animal interaction test, skin lesions and pig growth

To confirm the effect of enrichment on pig welfare status, behavioural observations, human animal relation test and skin lesion scores were assessed. Body weight  was also recorded to monitor the growth of piglets.

**Fig. 1:**
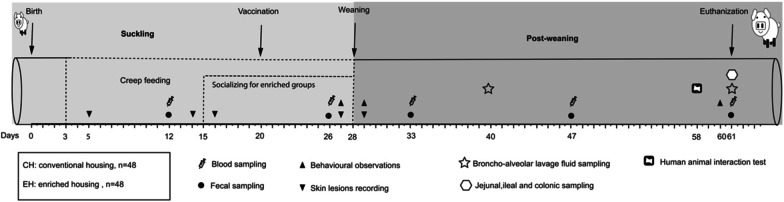
Experimental set-up. Pigs were housed in either conventional (CH) or enriched (EH) condition from birth till the end of the experiment period (day 61). Behavioural observations, human animal interaction test and skin lesion scores were assessed as a proxy for stress. Blood and broncho-alveolar lavage fluid were collected to measure leukocytes. In addition, blood samples were evaluated for cell blood count and re-stimulated with mitogens. Faeces and luminal digesta were collected and processed for 16S rRNA gene sequencing

Frequencies of the behaviours listed in the ethogram described previously [3] with a small modification were recorded on the days before and after weaning (day 27 and 29) and at the end of the rearing period (day 60) (Fig. 1). Behaviours in our ethogram consisted of aggression (uni- or bilateral fighting by chasing head knocking, with or without biting and/or pushing), mounting (standing on hind legs with front legs on pen mate), manipulating pen or pen mate (nibling, suckling or chewing on any other body part of piglets or sow or pen components, belly nosing), social behaviour (touching or sniffing any body part of a pen mate), playing (fast running around pen, rolling and shaking objects) as well as rooting. A new bout was scored when the pig stopped the behaviour for more than 2 s. On each observation day, all pens were continuously observed in a random order for a duration of 5 min per pen, twice in the morning and twice in the afternoon. The amount of behavioural bouts was scored per pig during the five minutes. Pen averages per 5 min were calculated and behaviours were expressed as average frequencies per pig per 5 min. Frequencies of behaviours that might be indicative of poor welfare (aggression, mounting, manipulating pen or pen mate) were pooled to one total score and referred to as ‘negative behaviour’. Behaviours that might be indicative of good welfare (social behaviour, play and rooting) were pooled to one total score, further referred to as ‘positive behaviour’.

On days 5, 14, 16 (before and after early socialisation), 27 and 29 (before and after weaning) (Fig. 1), skin lesions at the front (head, neck shoulders and front legs), middle (flanks and back) and rear (rump, hind legs, tail) were counted and categorized as a proxy for aggressive behaviour [19]. For each body region, the number and severity of lesions was differentiated using scores from 0–4 as follows (modified from [20]): 0: No lesions; 1: < 5 superficial lesions; 2: 5–10 superficial lesions or < 5 deep lesions; 3: 10–15 superficial lesions or 5–10 deep lesions; 4: > 15 superficial lesions or > 10 deep lesions. Lesions were scored per pig and averaged per pen and per day for further analysis.

Human Animal Relation Test (HART) was performed at day 58 (Fig. 1). An unfamiliar observer in white overall entered each pen and kneeled for 3 min. Piglets were scored 1 to 3 according to possibility to have physical contact. 1: nose contact was possible, and the head, neck or body could be touched; 2: nose contact was possible, but no contact with head or body; 3: no (nose) contact possible. Percentages of number of piglets per score were calculated.

Body weight (BW) of the piglets was measured weekly from the day of parturition until the end of the experiment.

### Selection of the piglets and sampling for further analysis

Per pen five piglets were randomly selected, balanced for sex, thus having 20 piglets per housing condition. From these piglets, heparinized blood samples were collected via jugular vein puncture on seven different days (days 12, 26, 33, 47 and 61) (Fig. 1), and were used for total count of white blood cells (WBC) and other haematology parameters. Out of these selected animals, two piglets per pen were then randomly selected for broncho-alveolar lavage fluid (BALF) sampling on day 40 (balanced for sex, 8 piglets per housing condition). Out of the 40 selected piglets, two other piglets per pen (balanced for sex, 8 piglets per housing condition) were randomly selected and kept for heparinized whole blood samples (at days 26, 47 and 61), and these blood samples were used for FACS analysis by flow cytometer. With the same selection method, 16 piglets (balanced for sex, 8 piglets per housing condition) were selected and kept for faecal sampling on the same seven days (days 12, 26, 33, 47 and 61) (Fig. 1). At the end of the investigation period (day 61), these 16 piglets selected for faecal sampling were euthanized, and then jejunal, ileal colonic digesta, as well as BALF were collected (Fig. 1). Furthermore, the whole blood samples were also used for the whole blood stimulation assay (on day 26 only).

Heparinized blood samples were collected via jugular vein puncture and properly stored for corresponding analysis. Faecal samples were taken directly from the rectum of piglets, and luminal digesta samples were collected and stored on dry ice, and then shipped to the lab and stored at − 80 ℃ for further analysis. BALF was obtained from living animals via the tracheal tube from the caudal lobe on day 40 and on day 61 during necropsy from the right cranial lung lobe. On day 40, the selected piglets were anesthetized using Zoletil (4 mg/kg) and Xylazine (2 mg/kg). During lavage, the pigs were held in ventral recumbency. A standard silicone tube with round tip (diameter of 4 mm) was inserted through the larynx into the trachea. When the catheter could not be inserted any further and reached a wedged position, it was pulled back for 0.5 cm and then 30 ml of PBS (phosphate buffered saline) kept at body temperature was slowly injected into the catheter. After 1 min the fluid was aspirated with a syringe and another 15 ml of PBS was injected into the catheter and aspirated after 1 min. The total aspirated amount of PBS per pig was between 20–35 ml. During necropsy, the BALF was obtained as previously described by van Dixhoorn, et al. 2016 [3], followed by isolation and phenotypic characterization of intra alveolar lymphocytes, granulocytes and monocyte/macrophages as described before [3].

### Blood and broncho-alveolar lavage fluid (BALF) analysis

The total count of white blood cells (WBC) and other haematology parameters were analysed on all blood sampling days with a haematology analyser (blood cell counter Sysmex pocH-100 iV diff, Kobe, Japan). The following variables were evaluated in this study: haemoglobin (Hb), haematocrit (Ht), platelet (Plt), red blood cell counts (RBC), mean corpuscular volume (MCV), and WBCs were counted and differentiated in WBC_large cell ratio (W_lcr; non-lymphocytes, non-neutrophils), WBC_mid cell ratio (W_mcr; neutrophils) and WBC_small cell ratio (W_scr; lymphocytes, monocytes).

Phenotyping of neutrophils (WBC mid cell ratio) and lymphocytes/monocytes was performed in whole blood samples collected on days 26, 47 and 61 and BALF samples on days 40 and 61 by flow cytometry as previously described by van Dixhoorn, et al. 2016 [3]. In brief, blood and BALF cells were incubated either with a primary antibody-mixture of three monoclonal antibodies (mAb) against CD3 (clone PPT3, isotype IgG1, Southern Biotech), CD4 (clone 74-12-4, isotype IgG2b, Southern Biotech), CD8 (clone 76-2-11, isotype IgG2a, Southern Biotech) for triple labelling, or for single labelled with mAb against CD172a (clone 74-2215, IgG2b, VMRD) or CD21 (clone BB6-11C9.6, isotype IgG1, Southern Biotech) for blood cells only, or CD14 (clone MIL2, isotype IgG2b, BioScource) and TLR4 (Isotype IgM, gift by J. Dominguez) for BALF cells only. The following combinations of secondary antibodies (SoutherBioTech, US) and fluorochromes were used: IgG1-APC, IgG2b-FITC and IgG2a-PE. The detailed information about antibody panels is available in Additional file 1: Table S1. Flow cytometric analyses were performed with a FACS flow cytometer (FACSVERSE™ (BD Biosciences) and data were analysed with Flowio™ software version10.0 [21] according to their antigen marker profile. The WBC’s and broncho-alveolar cell populations are presented as percentage of the total cell population [21]. The following immune cells were identified based on forward-scatter (FCS) versus sideward scatter (SSC) diagram as previously described [22, 23]. The T lymphocytes subpopulation in both blood and BALF cells were identified, the total T cells as referred to CD3^+^, cytotoxic T cells as CD3^+^CD4^−^CD8^+^, natural killer (NK) cells as CD3^−^CD4^−^CD8^+^, T helper (Th) cells as CD3^+^CD4^+^CD8^−^, memory Th cells as CD3^+^CD4^+^CD8^+^. The following possible populations of macrophages in BALF were calculated separately: all macrophages with expression of CD172^+^ or TLR4^+^ or CD14^+^, and with the following co-expressions: CD14^+^TLR4^+^, CD14^+^TLR4^−^, CD14^−^TLR4^+^, CD172^+^TLR4^+^, CD172^+^TLR4^−^, CD172^−^TLR4^+^, CD172^+^CD14^+^. B cells in blood were defined as CD21^+^ and granulocytes, monocytes were distinguished as SSC^high^ CD172a^+^ and SSC^low^CD172a^+^, respectively. While the granulocytes in BALF were identified by the specific marker.

Furthermore, the whole blood (obtained on day 26, eight piglets per housing condition) was re-stimulated with lipopolysaccharide (LPS), and levels of cytokine production (IL1ß, IL-6, TNF-α) were quantified by ELISA. The results of the WBC counting were used to dilute the blood with RPMI 1640 medium Glutamax with 5% foetal calf serum and 1% penicillin/streptomycin to obtain a WBC concentration of 5 × 10^6^ cell/ml. Then 0.5 ml of such diluted blood was transferred to 48 wells plates, and cells were either stimulated by adding 50 µl LPS/PBS solution (*Escherichia coli*, Sigma-Aldrich Chemie N.V., NL), i.e., 1 µg LPS/ml or 50 µl PBS only. Cells were incubated for 20 h before culture medium was harvested and stored at − 80 °C until performing cytokine analysis. The cytokines were determined by using the commercially available ELISA kits Duoset™ (R&D Systems) for IL1ß and IL- 6, and ELISA kit Quantikine™ (R&D Systems) for the analysis of TNF-α according to the manufacturer’s instructions.

### Microbiota profiling

The composition of the faecal and gut luminal microbiota of piglets was determined by barcoded 16S ribosomal RNA (rRNA) gene sequencing using Illumina Hiseq2500. DNA was extracted from faecal and luminal samples by repeated bead beating [24] and subsequent DNA purification. DNA was purified using an automated system, the Maxwell® 16 Research Instrument (Promega, Madison, USA) as previously described [25]. Purified DNA with a concentration of 20 ng/ul was used as a template for triplicate PCR amplifications with primers BSF784/R1064 targeting the V5-V6 region of the bacterial 16S rRNA gene [26]. Detailed information about the PCR reaction and cycling conditions can be found elsewhere [27]. Triplicate PCR products from each sample were pooled and purified by using the CleanPCR kit (Clean NA, the Netherlands). Concentration of purified DNA amplicons was determined with the Qubit BR dsDNA assay kit (Invitrogen by Thermo Fisher Scientific, Eugene, OR, USA). Finally, equimolar amounts of purified PCR products were pooled into libraries and sent for Illumina Hiseq sequencing with a read length of 2 × 150 bp (GATC-Biotech, Konstanz, Germany, now part of Eurofins Genomics Germany GmbH). Raw sequence data was first processed using the NG-Tax pipeline using default settings [28] and assigned to amplicon sequence variants (ASVs) using the Silva128 reference dataset [29]. ASVs with a relative abundance lower than 0.1% in a given sample were excluded on a per-sample basis.

One animal in the EH group ileal digesta could not be sampled because of an empty intestine, and DNA isolation failed for four jejunal samples from CH pigs. Furthermore, one faecal sample from one of the CH pigs at day 47 was outside an overall 95% confidence interval on weighted Unifrac based unconstrained principal coordinate analysis (PCoA). This outlier was discarded from the dataset used for downstream analysis.

### Statistical analysis

For non-microbial data, statistical analyses were performed with SAS (SAS 9.3, SAS Institute Inc.). For all data except the HART, mixed linear models were used with pen as random effect. Behaviour and skin lesions were analysed with the calculated average pen scores per pig as behaviours and skin lesions of individual pigs within a group are not independent. All other variables were analysed with pig nested in the housing regime as observational unit. Repeated measurements of variables on different days (BW, haematology parameters) were analysed using a linear mixed model for repeated measures analyses. Significant interactions were further analysed with post hoc pairwise comparison using the difference of the least square means and adjusted using Tukey correction. Results are presented as means ± SEM. If needed, variables were square root transformed to obtain normally distributed residuals. To analyse the effect of housing condition on HART, an ordinal logistic regression analysis was performed with the cumulative logit model in SAS, a graphical display of the odds ratios is presented.

For microbial sequence data analysis, ASV read counts were first normalized to relative abundance and were further analyzed using the R environment (version R-3.6.1) [30]. The microbial composition was analyzed at different taxonomic levels, ranging from ASV and genus to phylum level. Alpha diversity was determined at ASV level using packages *picante* [31] and *microbiome* [32]. Nonparametric Wilcoxon rank sum test was applied to assess whether the alpha diversity and the relative abundance of specific microbial taxa were significantly different between groups. Beta diversity at ASV level was computed based on pairwise sample Bray–Curtis dissimilarity [33], and weighted UniFrac [34] as well as unweighted UniFrac distance metrics [35], and the results were further visualized by PCoA using the *phyloseq* R package [36]. Bray–Curtis dissimilarity reflects microbial composition with ASVs representing independent observations, while weighted and unweighted UniFrac also take the phylogenetic relationships among ASVs into account. Furthermore, weighted UniFrac takes relative abundance of ASVs into account, whereas unweighted UniFrac only considers their presence or absence, thereby placing emphasis on less abundant taxa. Permutational multivariate analysis of variance (PERMANOVA) was performed using the Adonis’ function in the *vegan* package [37] to test the significance of differences in overall microbial composition. The linear discriminant analysis effect size (LEfSe) method [38] was chosen to identify biomarkers characterizing differences between groups. Principal response curves (PRC) analysis was performed to summarize differences between groups in microbial composition over time based on above-mentioned dissimilarity and distance metrics [39]. Furthermore, we performed distance-based redundancy analysis (db-RDA) to assess the multivariate effects of environmental variables on faecal and gut luminal microbiota composition using the capscale function from the *vegan* package [37]. For faecal microbiota, parameters included were housing, BW, sex as well as the following variables of haematological profile: Hb, Ht, Plt, RBC, MCV, W_lcr, W_mcr and W_scr. Missing values of environmental variables were imputed using the K-nearest neighbour algorithm as implemented in the *vim* package [40]. Data was evaluated before and after weaning, and time as the main driving factor was taken as conditional effect in the model. Forward and reverse automatic stepwise model selection using permutation tests with the ordistep function was performed to determine which set of environmental variables was responsible for the most parsimonious model. For gut luminal microbiota, we included variables of housing, sex, T cells and macrophages derived from BALF cells, including Leukocytes, the total T cells, cytotoxic T cells, NK cells, Th cells, memory Th cells, and macrophage subpopulations. Data was separately evaluated according to gut location and the same method as aforementioned for parsimonious model selection. All *p* values from multiple testing were corrected with a false discovery rate according to the procedure by Benjamini–Hochberg [41]. Differences were expressed as significant if adjusted *p* ≤ 0.05 or tendencies if 0.05 < *p* < 0.1.

## Results

### The effect of housing on animal behaviours, human animal relation test and skin score

We first assessed whether the different housing conditions affected piglet behaviour on three days throughout the experiment, i.e., days 27, 29, and 60. Pigs in the EH group showed higher frequency (freq.) of positive behaviour than CH pigs, with a significant difference on day 27 (CH: 0.004 ± 0.00 freq./5 min, EH: 0.15 ± 0.06 freq./5 min, *p* < 0.05) and tendencies on day 29 (CH: 0.3 ± 0.2 freq./5 min, EH: 2.3 ± 1.0 freq./5 min, *p* = 0.07) and day 60 (CH: 3.5 ± 0.8 freq./5 min, EH: 5.8 ± 0.9 freq./5 min, *p* = 0.07). In turn, CH pigs demonstrated higher frequency of negative behaviour (CH: 8.1 ± 1.4 freq./5 min, EH: 3.7 ± 1.7 freq./5 min, *p* = 0.05) than their EH counterparts on day 29.

**Fig. 2:**
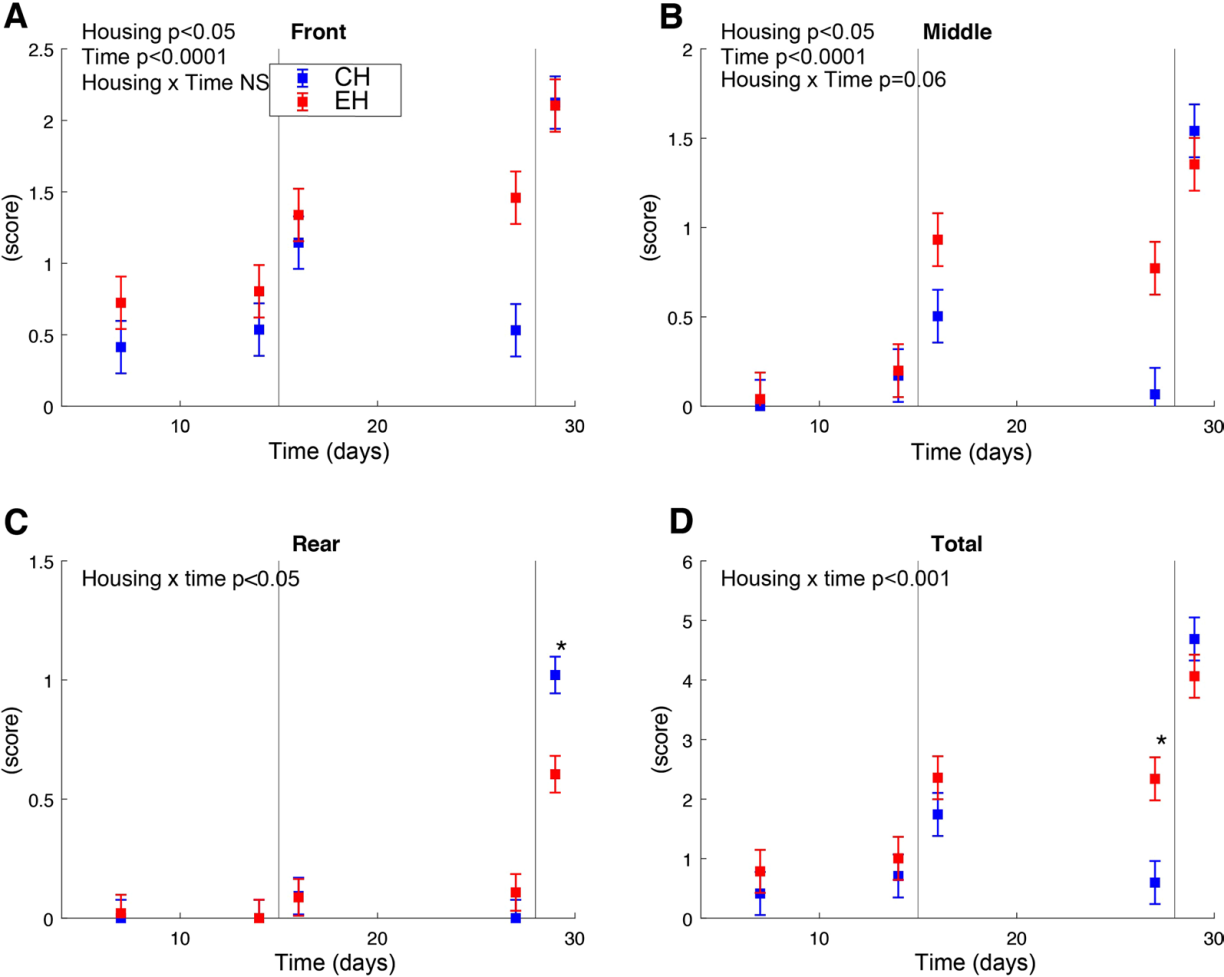
Skin lesion scores (mean of a four-point scale ± standard error). **A**–**C** Scores from the front, middle and rear of pigs, respectively. **D** Shows all scores. Blue and red colours represent conventional housing (CH) and enriched housing (EH), respectively. ‘*’ indicates a significant difference for CH versus EH pigs (post hoc comparison, *p* < 0.05). The vertical lines at day 15 and 28 indicate socialisation in the EH pigs and weaning for both groups

EH pigs had overall higher skin lesion scores at the front and middle part of the body (*p* < 0.05, Fig. 2A, B). For skin lesions at the rear and the total score of the whole body, a housing × time interaction was significant (*p* < 0.05 and *p* < 0.001, Fig. 2 C, D). Post hoc comparison showed that scores for lesions at the rear were higher in the CH group after weaning on day 29 as compared to the EH pigs (*p* < 0.05, Fig. 2C). Before weaning (day 27) the total lesion scores in the CH pigs were lower as compared to the EH pigs (*p* < 0.001, Fig. 2D). Furthermore, Human Animal Relation Test (HART) was performed to assess the piglets’ fear response to humans. The nose of 37 (out of 48) EH pigs could be touched and of those 16 pigs could be also touched on neck and head. Of the CH pigs the nose of only 21 pigs could be touched, and none of them was willing to be touched on the head or neck. In total, 11 pigs could not be touched in the EH group as opposed to 27 in CH group. With the odds of 0.21 (*p* < 0.05), the CH pigs were less likely to score a low score (fast encounter to human) as compared to the EH pigs. Finally, no differences in body weight were observed between both housing conditions in this study (Additional file 1: Fig. S1A).

### The effect of enrichments on blood cell counts, whole blood and BALF immune cell phenotyping, and whole blood stimulation assay

**Fig. 3:**
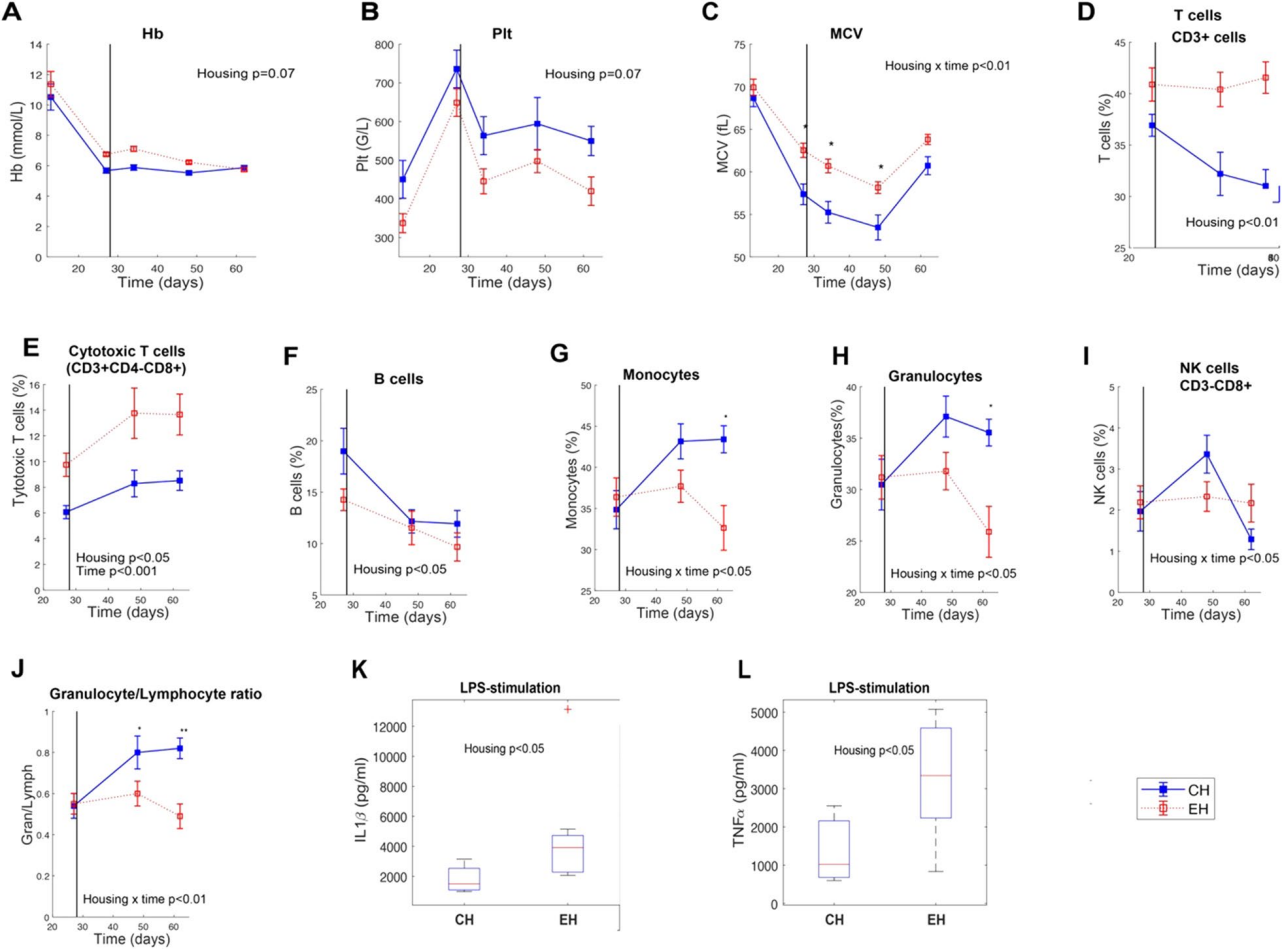
The effect of housing condition on cell blood count, leukocyte subpopulations and whole blood stimulation assay. **A**–**C** represent haemoglobin (Hb), platelet (Plt) and mean corpuscular volume (MCV), respectively. **D**–**I** sequentially show the leukocyte subpopulations of the total T cells, cytotoxic T cell, B cells, monocytes, granulocytes and NK cells. **J** expresses the ratio of granulocyte/lymphocyte (Gran/Lymph). **K**, **L** exhibits the difference between both housing conditions in the level of IL1ß and TNF-α after lipopolysaccharide (LPS) challenge for the whole blood stimulation assay. ‘*’ and ‘**’ indicate significant differences for conventional housing (CH) vs. enriched housing (EH) pigs (post hoc comparison, *p < 0.05, **p < 0.01)

A tendency for housing effect was observed for haemoglobin and platelet where EH pigs tended to have a higher level of Hb (*p* = 0.07) and a lower value of Plt (*p* = 0.07) (Fig. 3A, B). A significant housing × time interaction (*p* < 0.01) was observed in the MCV value where the level was higher in EH pigs. Post hoc comparison showed significant differences between both housing conditions on days 26, 33 and 47 (*p* < 0.05) (Fig. 3C). Among the other haematological variables, no differences between CH and EH pigs were identified at the investigated days. With respect to peripheral blood leukocyte subpopulations, EH pigs had a significantly higher percentage of the total T cells (*p* < 0.01), cytotoxic T cells (*p* < 0.05), and lower levels of B cells (*p* < 0.05) (Fig. 3D–F). A significant interaction of housing × time (*p* < 0.05) was found for monocytes, granulocytes and NK cells. Post hoc analysis revealed that percentages of monocytes and granulocytes were significantly lower in EH pigs than in CH pigs on day 61, whereas no differences were found for NK cells between EH and CH pigs by post hoc comparison (Fig. 3G–I). Over and above that, a significant housing × time interaction regarding to the ratio of granulocytes to lymphocytes was found. Post hoc analysis further disclosed that the ratio was significantly higher in CH pigs compared to EH at days 47 and 61 (Fig. 3J). In term of Th cells and memory Th cells in blood, no differences were observed between CH and EH pigs (Additional file 1: Fig. S1B&C).

LPS stimulation of blood cells resulted in increased secretions of IL1ß and TNF-α in EH pigs than in CH pigs (*p* < 0.05) (Fig. 3L, M). For the BALF cell populations, only memory Th cells showed a higher proportion in EH pigs than in CH pigs (*p* = 0.05) at day 61 (Additional file 1: Fig. S1D). Housing did not significantly affect any other cell population in BALF at day 40 or 61.

### Dynamic effect of enrichments on piglet gut microbiota colonization during early life development

In addition to the comparison of behaviour and immune system development, we also evaluated whether housing and social enrichment would affect the development of the gut microbiota. To this end, we determined microbial composition in faecal samples as well as jejunal, ileal, and colonic digesta samples taken at sacrifice.

A first general PCoA, irrespective of the enrichments, showed that the main driver for faecal microbiota development was time (age) together with diet, irrespective of the used metric, i.e., weighted/unweighted UniFrac distance or Bray–Curtis dissimilarity (Additional file 1: Fig. S2A-C). Alpha diversity of pig faecal microbiota, based on phylogenetic diversity, observed richness and inverse Simpson indices, significantly increased from day 12 to day 26 prior to weaning, whereas we observed a small but significant decrease from day 26 to day 33 of phylogenetic diversity and inverse Simpson (Additional file 1: Fig. S2D-F). During the remainder of the investigated period, alpha diversity remained relatively stable. With respect to the luminal microbiota, intestinal location was the main factor driving observed differences in microbial composition, both with respect to alpha- as well as beta-diversity (Additional file 1: Fig. S3). To this end, the colonic microbiota was most distinct, whereas the two small intestinal locations, i.e., jejunum and ileum, were more similar in microbial composition.

**Fig. 4:**
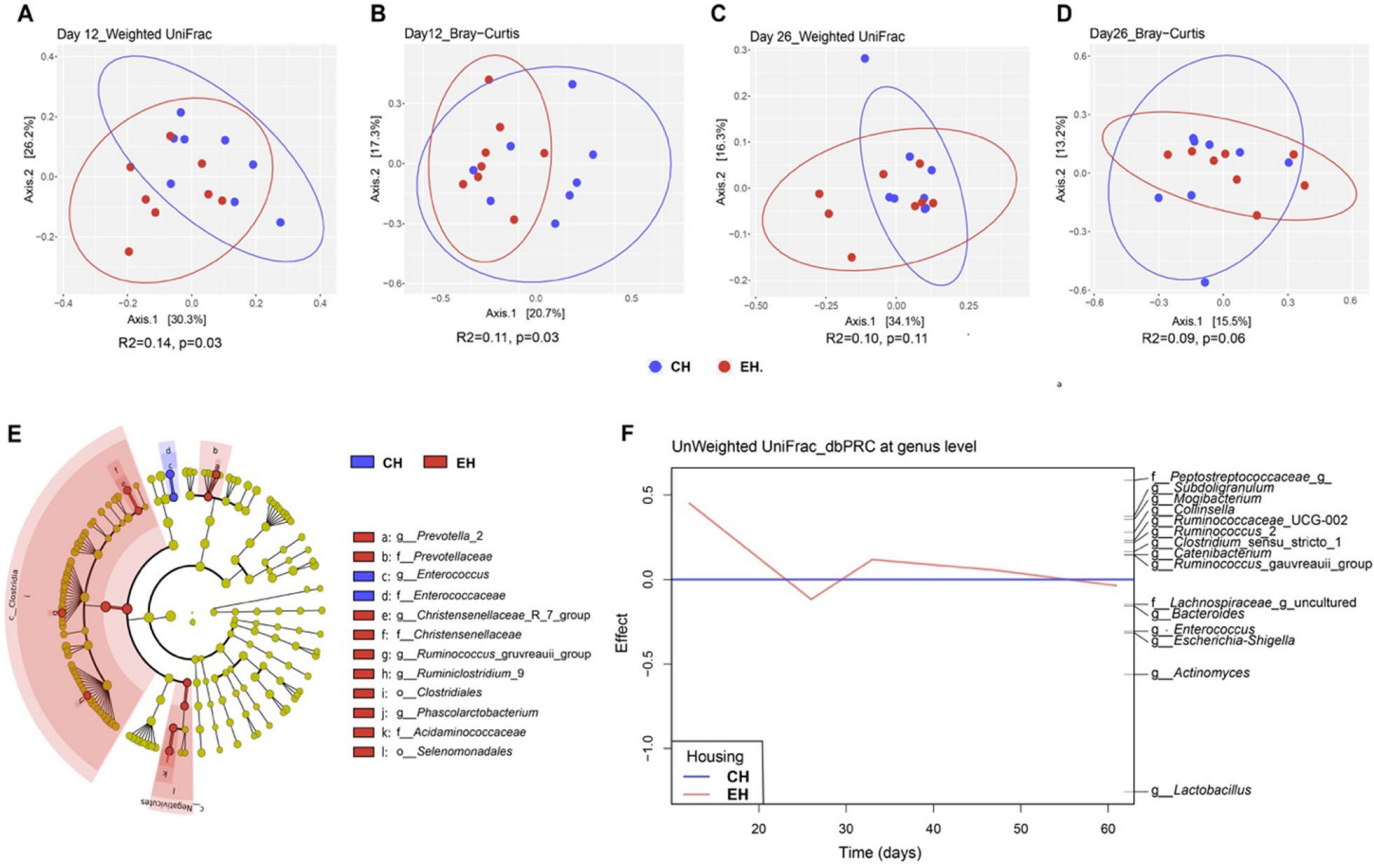
The effect of housing condition on microbiota colonization. Principal coordinate analysis (PCoA) plots based on pairwise weighted UniFrac distance and Bray–Curtis dissimilarity matrices using data from day 12 (**A**, **B**) and day 26 (**C**, **D**) at amplicon sequence variant (ASV)-level. Significance of the difference between conventional housing (CH) and enriched housing (EH) was assessed using PERMANOVA. **E** Cladogram of taxa differentially abundant between CH and EH pigs on day12 according to LEfSe. The color indicates the difference between both housing types in which the taxa were more abundant and the radius of each shaded sector is corresponding to the relative abundance. **F** Unweighted UniFrac based principal response curve (dbPRC) summarizing the multivariate response of EH versus CH at genus level over time. Genera with large deviations between CH and EH have high effect while taxa equally present in CH and EH have zero effect

PCoA showed that housing type mainly influenced piglet gut microbiota colonization before weaning. Pig microbial composition was significantly different between CH and EH houses based on weighted or unweighted UniFrac distance and Bray–Curtis dissimilarity matrices on day 12 (*p* < 0.05) (Fig. 4A, B, Additional file 1: Fig. S4A). On day 26, piglet faecal microbiota did not differ significantly between both housing conditions, but PERMANOVA revealed a tendency using Bray–Curtis (*p* = 0.06) (Fig. 4D). In addition, we observed a significantly larger inter-individual variation within the EH group in comparison with CH pigs based on weighted UniFrac distances on day 26 (*p* = 0.05) (Additional file 1: Fig. S4B). In order to identify individual taxa most strongly associated to the observed differences between animals raised in EH or CH, we used the LEfSe algorithm. This revealed six differences at the genus level, four at family level and two differences at the order level for CH vs. EH pigs on day 12 (Fig. 4E). At the genus level, *Prevotella*_2, *Christensenellaceae*_R_7_group, *Ruminococcus_gauvreauii*_group, *Ruminiclostridium*_9 and *Phascolarctobacterium* showed higher relative abundance in EH pigs as compared to CH pigs, whereas *Enterococcus* was decreased. At higher taxonomic levels, the families *Prevotellaceae, Christensenellaceae* and *Acidaminococcaceae* and orders *Clostridiales* and *Selenomonadales* showed higher relative abundance in piglets subjected to enriched housing whereas *Enterococcaceae* was more abundant in CH pigs. No significant difference was observed in faecal microbiota composition between both housing conditions after weaning as assessed by PCoA and PERMANOVA. Alpha diversity did not differ on any of the investigated days (Additional file 1: Fig. S5A-C). To investigate more specifically whether and to what extent exposure to different housing environments affected the development of piglet faecal microbiota community at genus level throughout time, we performed PRC analysis. Using unweighted UniFrac distances, which provided stronger separation than corresponding weighted UniFrac distances, 4.98% of the observed variation in microbial composition was explained by the interaction of housing × time (*p* = 0.08) (Fig. 4F). This PRC model identified a significant housing effect on day 12 (*p* = 0.007), reflecting the above-mentioned unconstrained ordination analysis that showed that the effect of enrichments was most pronounced during pre-weaning, and especially on day 12. Based on the PRC model, we observed that differences between both groups were most strongly related to the presence of *Lactobacillus* in CH pigs and the presence of a not further annotated genus-level group with the *Peptostreptoccaceae* in the EH group. We further plotted the top 15 genera over time that showed the best fit with the first axis from the PRC model and evaluated differences in their relative abundance between groups per timepoint. Three out of these 15 genera had a significantly higher relative abundance in EH at different timepoints. Among these, *Ruminococcus gauvreauii*_group (*p* = 0.02) showed a significantly higher relative abundance in animals of the EH group on day 12 (Additional file 1: Fig. S6A), confirming the LEfSe analysis. Furthermore, *Lachnospiraceae*_g_uncultured (*p* = 0.03; Additional file 1: Fig. S6B) and *Catenibacterium* (*p* = 0.04; Additional file 1: Fig. S6C) were significantly more abundant in piglets of the EH group on day 26 and day 33, respectively.

### Association between housing and host-related parameters with pig faecal microbiota

**Fig. 5:**
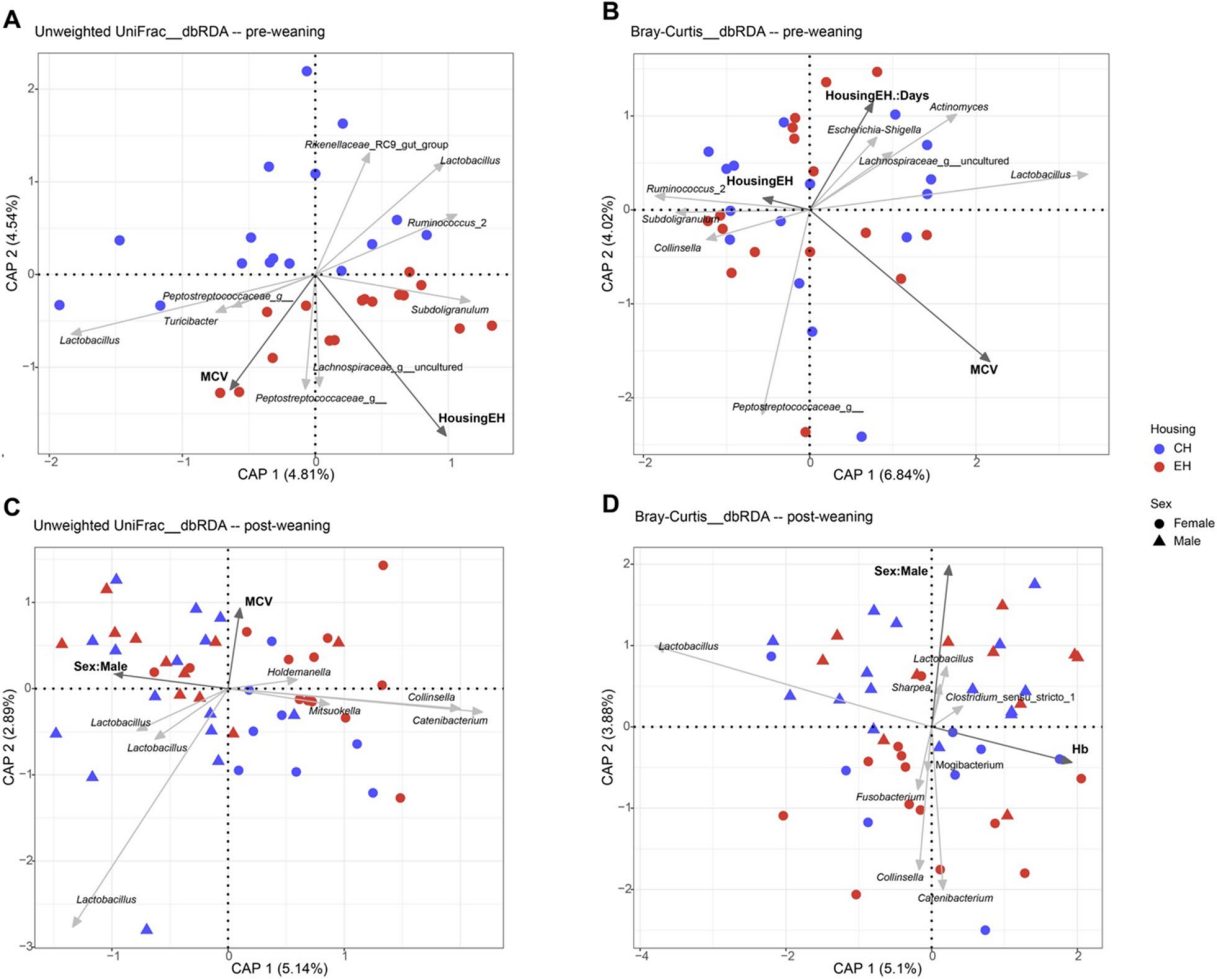
Distance-based redundancy analysis (db-RDA) triplot showing the association between faecal microbiota variation and environmental variables. **A**, **B** Focusing on samples from pre-weaning (day 12 and day 26), **C**, **D** samples from post-weaning (day 33, day 47 and day 61). Colours represent different housing conditions (conventional housing: CH; enriched housing: EH) and shapes show different sex. Dark grey arrows indicate environmental variables and light grey arrows ASVs for which the model provided the best fit for the observed variation. The factor time (Days) was taken as the conditional variable in db-RDA analysis

During pre-weaning, we did not observe any set of variables significantly contributing to explaining the observed microbiota variation using weighted UniFrac based db-RDA, except for the interaction of housing and time (*p* = 0.014) (Additional file 1: Fig. S7A). In contrast, unweighted UniFrac based db-RDA illustrated that faecal microbiota significantly differed between both housing conditions (*p* = 0.041), and MCV also partly explained microbial variation (*p* = 0.042) (Fig. 5A). EH correlated with the presence of *Lachnospiraceae*_g_uncultured, *Peptostreptoccaceae*_g_*_*and *Subdoligranulum* ASVs*.* The continuous variable MCV was positively correlated with the presence of *Lachnospiraceae*_g_uncultured, *Turicibacter* and two *Peptostreptoccaceae*_g__ ASVs and inversely related to the presence of *Ruminococcus*_2 and *Rikenellaceae*_RC9_gut_group ASVs. Intriguingly, MCV was positively and negatively correlated with the presence of two *Lactobacillus* ASVs, respectively. Using Bray–Curtis based db-RDA, microbial variation was partly explained by MCV (*p* = 0.006) and the interaction of EH × time (*p* = 0.015) (Fig. 5B). ASVs from genera *Collinsella* and *Subdoligranulum* were negatively correlated with MCV. Consistent with the presence of *Ruminococcus_2* ASV in unweighted UniFrac*,* MCV was also inversely correlated with this ASV. During post weaning, the factor of housing no longer significantly drove the microbial variation based on any of the distance matrices, confirming by abovementioned PCoA and PRC analyses. Hb significantly contributed to explaining the microbial variation (*p* = 0.009) and mainly inversely correlated with the relative abundance of one *Lactobacillus* ASV using weighted UniFrac based dbRDA, while sex showed a tendency (*p* = 0.08) with male being mainly inversely correlated with the relative abundance of *Collinsella* (Additional file 1: Fig. S7B). With unweighted UniFrac, sex significantly contributed to the microbial variation (*p* = 0.003) (Fig. 5C). Male was positively correlated with the presence of three *Lactobacillus* ASVs and inversely correlated with the presence of ASVs from the genera *Catenibacterium*, *Collinsella, Mitsuokella* and *Holdemanella.* The variable MCV demonstrated a tendency to significantly contribute to the microbial variation (*p* = 0.08) and was mainly inversely correlated with the presence of three *Lactobacillus* ASVs that were positively correlated with male. Using Bray–Curtis based dbRDA, microbial variation was significantly explained by Hb (*p* = 0.004) and sex (*p* = 0.017) (Fig. 5D). Hb was mainly inversely correlated with one *Lactobacillus* ASV. Male correlated with ASVs from the genera *Lactobacillus*, *Sharpea* as well as *Clostridium*_sensu_stricto_1, while it was inversely correlated with ASVs from genera *Catenibacterium*, *Collinsella*, *Fusobacterium* and *Mogibacterium.*

### Gut location-dependent effect of housing condition and sex on luminal microbiota

**Fig. 6:**
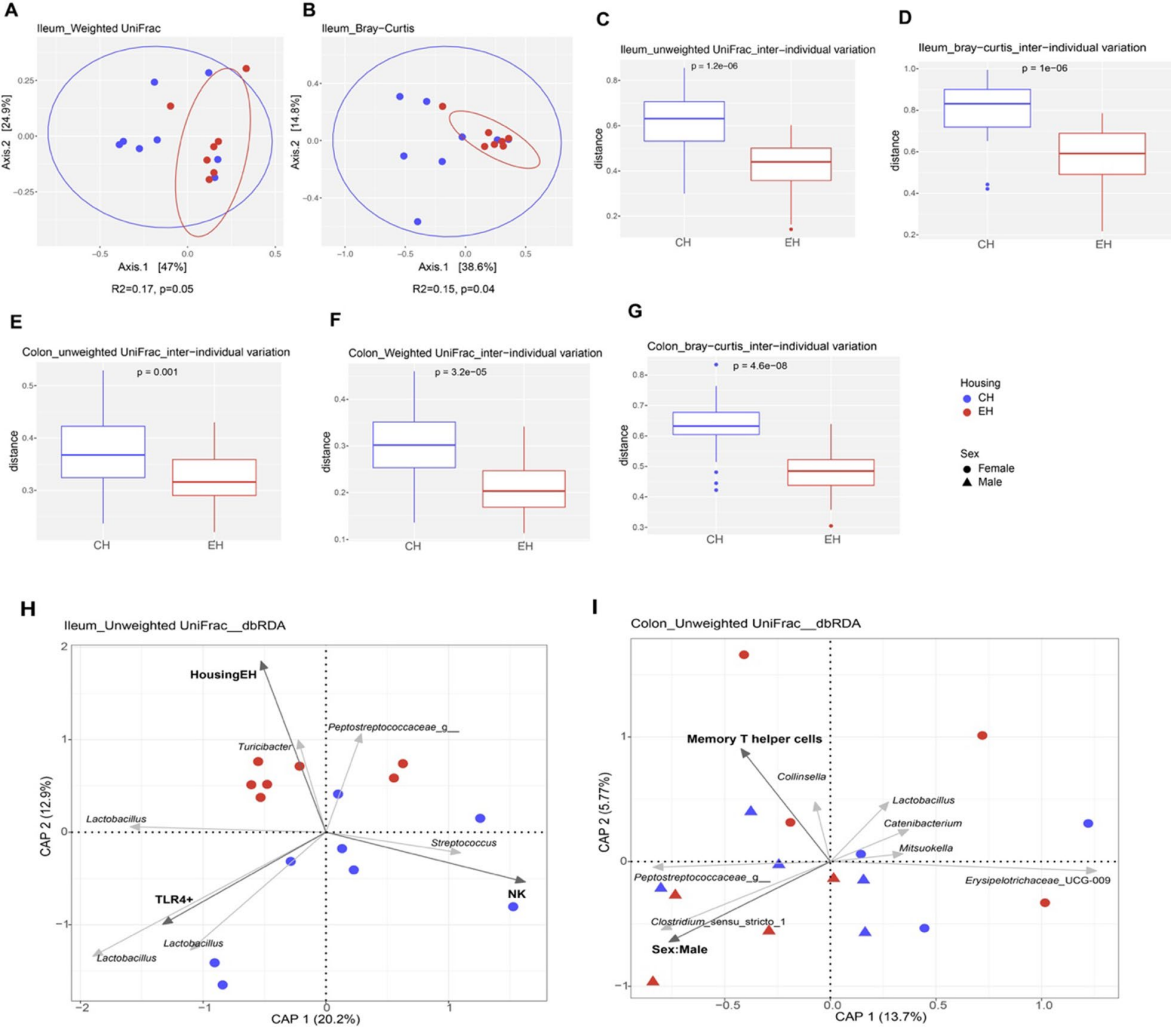
The effect of housing condition on ileal and colonic microbiota composition. Based on either unweighted or weighted UniFrac pairwise distance or Bray–Curtis pairwise dissimilarity at amplicon sequence variant-level, **A**, **B** Microbial dissimilarities of ileal digesta between CH and EH pigs were assessed by principal coordinate analysis (PCoA) and PERMANOVA. **C**–**G** Differences in inter-individual variation for CH versus EH pigs in ileal and colonic microbiota composition was asessed by Wilcoxon rank sum test*.* Distance-based redundancy analysis (db-RDA) triplots show the association between microbiota variation and environmental variables, focusing on samples from ileum (**H**) and colon (**I**). Blue and red colours represent conventional housing (CH) and enriched housing (EH), respectively. Dark grey arrows indicate environmental variables and light grey arrows show best fitting ASVs

For luminal microbiota on day 61, housing condition mainly modulated pig ileal microbial composition. PCoA supported ileal microbiota being different between EH and CH pigs based on weighted UniFrac (*p* = 0.05) (Fig. 6A) and Bray–Curtis (*p* = 0.04) indices (Fig. 6B). LEfSe analysis further revealed several differentially abundant ileal taxa between CH and EH pigs (Additional file 1: Fig. S8). Bacteria *Clostridiaceae*_1, *Clostridium_sensu_stricto*_1, *Escherichia_Shigella*, *Enterobacteriales* and *Enterobacteriaceae* were more abundant in EH piglets, but the relative abundance of *Firmicutes* was higher in CH piglets*.* Besides, the inter-individual variation of ileal microbiota was larger in CH pigs than in the EH group using unweighted UniFrac (*p* = 1.2e-06) (Fig. 6C) and Bray–Curtis (*p* = 1e-06) (Fig. 6D). For colonic microbiota, CH pigs had significantly larger inter-individual microbial variation in comparison with EH pigs, based on weighted UniFrac (*p* = 3.2e-05) (Fig. 6E), unweighted UniFrac (*p* = 0.001) (Fig. 6F) and Bray–Curtis (*p* = 4.6e-08) (Fig. 6G).

For db-RDA analysis, we did not observe any variable significantly explaining either ileal or colonic microbiota variation using weighted UniFrac db-RDA. With unweighted UniFrac, db-RDA revealed that ileal microbial variation was explained by NK (*p* = 0.01), TLR4^+^ (*p* = 0.02) and housing (*p* = 0.05) (Fig. 6H). NK mainly correlated with the presence of a *Streptococcus* ASV and inversely correlated with the presence of a *Turicibacter* ASV as well a*s* three *Lactobacillus* ASVs*.* While the presence of those *Lactobacillus* ASVs was positively correlated with TLR4^+^, this variable was oppositely correlated with the presence of *Turicibacter and Peptostreptoccaceae*_g__ASVs that were correlated with enriched housing. For colonic microbiota composition, the variation was mainly explained by sex*.* Using unweighted UniFrac, sex slightly though significantly related to colonic microbiota variation *(p* = 0.04), and memory Th cells showed a tendency (*p* = 0.09) (Fig. 6I). Memory Th cells correlated with the presence of *Collinsella* and *Peptostreptoccaceae*_g__ASVs*.* Sex:Male correlated with the presence of *Peptostreptoccaceae*_g__and *Clostridium*_sensu_stricto_1 ASVs and inversely correlated with the presence of ASVs from genera *Collinsella, Lactobacillus, Catenibacterium, Mitsuokella* and *Erysipelotrichaceae*_UCG_009*.*

## Discussion

In a previous study we demonstrated that social and environmental enrichments applied from birth onwards significantly reduced the disease susceptibility to co-infection with PRRSV and *A. pleuropneumoniae* on day 44 after birth [3]. In addition, this previous study showed that pigs reared under enriched housing condition also showed a decrease of stress related behaviours around weaning compared to conventionally housed pigs [3]. The aim of the current study was to compare the effect of different housing conditions for pigs on the development and dynamics of gut microbiota colonization, systemic and local pulmonary immune cell composition. To the best of our knowledge, this is the first study that characterized the interplay between socially and environmentally enriched housing and the temporal dynamics of microbial composition, systemic and local pulmonary immune cell composition for pigs during the suckling period (day 12, 26) and nursery (day 33, 47, 61). Immune cell composition and stress related behaviours were different between the housing conditions, and these observations further contributed to the evidence that enriched rearing can positively influence pig immune competence and welfare [3, 12, 42]. In addition, the faecal microbiota was slightly but significantly affected by housing conditions during pre-weaning. This finding is in line with previous studies, where environmental conditions were shown to be an important factor driving gut microbiota [43–46]. After weaning the difference in faecal microbiota composition between CH and EH pigs became smaller, and only the ileal microbiota showed a tendency to differ between both housing conditions at the end of the investigated period.

As expected, behavioural assessment and skin lesion scores showed more positive behaviour, less aggression behaviour during the weaning transition and a less fearful human animal relation in pigs reared under enriched housing conditions. Hence, this study confirms previously observed effects of enrichment on the welfare of pigs [3, 42, 47, 48]. The increased time spent on positive behaviour, such as play, rooting and social interaction, was most probably due to the bedding substrates, extra toys, larger spaces and early socialization in EH pigs, while CH pigs showed higher levels of time spent on damaging, oral manipulation of pen or penmates, and aggression associated behaviour. The differentiation of pig behaviour was also reflected by skin lesions. Turner et al. [19] specified that skin lesions to the anterior and central regions of the body represented an engagement in reciprocal fighting, whilst the receipt of bullying (e.g., unilateral fighting or biting while the other piglet runs away) led to lesions accruing at posterior regions of the body. We observed that skin lesion scores were significantly higher in EH pigs at the front and middle during the suckling period whereas lesions were more prevalent in the rear of CH pigs after weaning. In other studies, the provision of enrichments without socialization during pre-weaning did not influence the number of skin lesions at weaning [18, 49]. It was also reported that social skills that socialized piglets obtained early in life may enable them to more quickly and efficiently establish a stable dominance hierarchy during aggressive encounters later in life (e.g., at weaning) [50–53]. This suggests that the stronger impact of weaning stress on skin injuries in the CH group as compared to EH pigs may largely be due to the absence of early-life socialization, in relation with conventional housing conditions. Accumulating evidence has pointed out that a poor human-animal relationship can lead to an animal’s fear of humans. Fearfulness is considered to have an adverse impact on animal welfare and production [54–56]. Our observation regarding HART showed EH pigs were more willing to interact with humans, indicating lower levels of fearfulness [56].

We also found that housing conditions exerted an effect on pigs’ immune status. Haematological parameters are important indicators of the health status in animals and play a crucial role in the diagnosis and prognosis of many diseases [57, 58]. We observed that Hb and MCV values were higher in EH pigs whereas CH pigs had higher levels of Plt in the present study. It should be noted that these values obtained from blood cell count were within expected ranges [59, 60]. Reduction in levels of MCV and Hb has been widely reported to be associated with anaemia [61–64]. Interestingly, previous evidence showed that PRRSV-infection can reduce the levels of Hb and MCV even in the absence of any clinical signs of anaemia [65–67]. These findings may be related to the previously observed reduction in severity of disease after co-infection with PRRSV and *A. pleuropneumoniae*, where pigs reared in enriched housing conditions showed a faster clearance of PRRSV RNA in blood serum and less interstitial pneumonia signs in the lungs [3]*.* Possibly an enriched environment might mitigate the potential risk of developing anaemia compared to conventional housing. For immune cells in peripheral blood, overall higher percentages of the total T cells and cytotoxic T cells were observed in EH pigs, and those pigs showed significantly lower levels of monocytes and granulocytes on day 61, as well as overall lower levels of B cell than CH pigs. It is interesting to note that the granulocyte/lymphocyte ratio was also significantly higher in CH pigs (day 47 and day 61). This increased ratio has been proposed as a marker for stress, such as surgical stress in humans [68] or transport stress in pigs [69]. Our observation is also in line with previous housing strategy related studies, where an increase in granulocyte/lymphocyte ratio was observed for pigs housed in conventional environments compared to those kept in enriched conditions [12, 70]. The impact on the granulocyte/lymphocyte ratio might thus complement our observations concerning pig welfare, such as skin lesions and human animal relationship, indicating that enriched rearing conditions reduced the stress levels in pigs. On day 26 prior to weaning, housing also exerted an immunomodulatory effect with respect to ex vivo secretion of IL1ß and TNF-α with higher levels in EH pigs as compared to CH pigs in response to the acute inflammatory stimulus LPS. LPS is known to stimulate immune cells to synthesize cytokines such as TNF-α, IL1ß and IL-6. These pro-inflammatory cytokine-mediated events are part of a general homeostatic reaction and thus serve as the first line of defence of the animal against infection [71, 72]. The lower TNF-α and IL1ß responses in CH pigs compared to EH pigs are probably not beneficial as this would likely indicate a slower or diminished response to LPS [71]. Thus, our observations suggest that the immune system of EH pigs prior to weaning is more capable of mounting an innate inflammatory response than that of CH pigs. Regarding to local pulmonary immune cells, only memory Th cells showed a tendency to have a higher value in EH pigs compared to CH pigs on day 61. Interestingly, the previous study [3] rather showed that pigs reared under conventional housing conditions had higher percentages of TLR4^+^ and CD172a^+^/TLR4 macrophages than pigs kept in enriched enrichments. This discrepancy could possibly be caused by differences in the timing of sampling and/or differences between SPF piglets that were used in the previous study and non-SPF pigs that were used in this study.

The structure of the faecal microbiota of CH and EH pigs significantly differed as early as day 12 with a decrease of *Enterococcus* and an increase in the relative abundance of a group of bacteria comprising members of the genus-level taxa *Prevotella*_2, *Christensenellaceae*_R_7_group, *Ruminococcus gauvreauii*_group, *Ruminiclostridium*_9 and *Phascolarctobacterium* in EH pigs. This may provide benefits to the host as these bacteria are known to be involved in the degradation of a wide range of plant derived polysaccharides and production of short chain fatty acids (SCFAs) [73–75]. Distance based PRC and RDA analyses showed that differences between both experimental groups with respect to faecal microbiota during pre-weaning and ileal microbiota were strongly related to the presence of *Lactobacillus* in CH pigs and the presence of *Peptostreptococcaceae*_g__in the EH pigs. Members of the genus *Lactobacillus* are associated with the degradation of lactose, which is one of the main components in porcine milk and transition diet [76–78]. The family *Peptostreptococcaceae* comprises a range of commensal bacteria in the gut and has been reported to be related to protein intake, as well as helping to maintain gut homeostasis [79, 80]. Moreover, *Lachnospiraceae*_g_uncultured and *Catenibacterium* were significantly more abundant in piglets of the EH group on day 26 and day 33, respectively. Members of the family *Lachnospiraceae* are well known for the degradation of fibre and production of SCFAs in the mammalian gut environment [81, 82]. Members of the genus *Catenibacterium* are Gram-positive anaerobic bacteria that utilize glucose to produce acetic, lactic, butyric and iso-butyric acids, and this genus was shown to be increased in relative abundance in pigs fed inulin and oat bran [83–85]. Those observations might indicate that exposure to socially and environmentally enriched housing accelerated the maturation of early-life microbiota composition towards plant-based diet consumption for EH pigs. This might be explained by the fact that the bedding materials used for the EH group (straw, moist peat and wood shavings) contained plant-derived compounds (carbohydrates, fibres) that were ingested by the animals through rooting behaviour that was significantly higher in EH piglets during suckling period. Additionally, there may be a potential effect of chronic stress on pig gut microbiota development. In the present study, pigs showed lower levels of proxies of stress in the EH group compared to the CH group according to the observations regarding to above mentioned welfare status and the granulocyte/lymphocyte ratio. It has also been reported in other studies that conventionally housed pigs suffered behavioural and physiological signs of chronic stress and had a more negative affective state [42, 86–88]. Evidence is growing from rodent and human studies that those adverse physiological and psychological factors can affect the gut microbiota that plays a key role in the bidirectional communication along the GBA via a network of neuronal, endocrine and immune cells through their metabolites (e.g., SCFAs) (reviewed by Molina-Torres et al. [16]). Microbiota and their metabolites have been further described as associated with the modulation of cognitive functioning and mental health in response to stress (reviewed by Vogel et al. [89]). Only speculative at this stage, the MGBA might also play an important role in response to conventional housing-induced chronic stress for pigs, although involvement of this axis has not yet been thoroughly evaluated in farm animals.

The faster maturation of pig gut microbiota observed here was also reported by Vo et al. [90], where only exposure to soil (from day 4 to day 13 postpartum) accelerated the maturation in pig gut microbial composition compared to conventionally reared piglets. However, there are also differences between studies with respect to the relation between microbiota colonization and rearing conditions with enrichments. This might be because pigs involved were at different ages and the specific characteristics of housing conditions used. Kubasova et al. [91] observed a moderate difference in faecal microbiota composition between sows kept under rearing conditions with or without enrichments. The microbiota of sows from the enriched unit covered with deep straw and more space during gestation was enriched in bacteria able to metabolize non-soluble polysaccharides, but these differences were not observed in the microbiota of their piglets (day 1 and day 4 postpartum). In contrast, Megahed et al. [92] proposed that a complex straw-based rearing ecosystem seemed not to provide optimal conditions for establishing a healthy microbial community in growing pigs, as they did not find marked differences in microbiota composition of animals reared in simple-slatted or complex straw-based conditions either at bronchus, ileum, colon, or faeces on day 164 post-weaning. This might be due to the fact that pigs were divided into the two experimental groups only starting from day 24 post-weaning until day 164, which may miss the “window of opportunity” to influence microbiota colonization during the early post-natal stage (pre-weaning period) [93]. Intriguingly, in our study, the inter-individual variation of ileal and colonic microbiota was significantly larger in CH pigs than in EH pigs on day 61, whereas this difference was not observed in faecal and jejunal microbiota at any timepoint, with the exception for day 26, where we observed that EH pigs had a larger inter-individual variation in faecal bacteria than CH pigs based on weighted UniFrac distance. Inter-individual variation is a common finding in gut microbiota reports [94]. Chen et al. [95] showed that the inter-individual variation between different piglets was significantly higher during suckling and markedly decreased upon weaning, suggesting that gut microbiota successively stabilizes and converges with age. In agreement with this, our observations demonstrated that continuous exposure to social and environmental enrichments resulted in a more homologous gut microbiota in EH pigs than in CH pigs at the end of nursery.

In the present study, in addition to the housing condition, there were also other host parameters that have been associated with gut microbiota development. Two haematology parameters, MCV and Hb, were significantly related to faecal microbial variation during pre-weaning and post-weaning, respectively. These two parameters have shown lower levels in CH pigs in our study and may reflect iron status. Mounting evidence specifies that iron status has a significant effect on pig gut microbiota composition and diversity (reviewed in [96]). To this end, we hypothesize that exposure to enriched conditions (e.g., soil peat) may supply extra iron for EH pigs, but further research is needed to unravel the mechanism underlying the observed association between MCV/Hb and gut microbiota. Furthermore, NK- and TLR4^+^ macrophage cells derived from BALF were significantly associated with ileal microbiota variation, although not with housing, and corresponding memory Th cells showed a tendency to contribute to colonic microbial variation on day 61. While it is difficult to pinpoint the mechanism underlying the correlation between gut microbiota and BALF cells, it is noteworthy that emerging data has indicated a bi-directional cross-talk between gut microbiota and the lungs [97]. Microorganisms and their metabolites not only influence gastro-intestinal immunity but also impact the distal organs (e.g. lung and brain) [98]. Hence it may be of interest for further studies to address the complex mechanisms that lie behind the associations for pigs in different housing regimes. Finally, sex was also shown to affect pig faecal microbial variation during post-weaning and colonic microbiota composition on day 61. Our observations were in line with previous pig studies that specified the effect of sex on gut microbiota structure [99, 100]. We postulate this sex effect may be caused by the hormonal and genetic difference as well as how males and females cope with stress [101].

### Conclusion

Collectively, data presented here support the notion that early social and environmental enrichment may be profitable for pigs not only by reducing damaging behaviours and changing immune competence, but also by accelerating the maturation of gut microbiota. This may contribute to improving health of pigs and reducing medicine (e.g., antibiotics) use. It would be interesting for future work to disentangle the different mechanisms by which environmental enrichment affects microbiota colonization and immunity in pigs and the subsequent consequences for animals’ welfare and health.

## Supplementary Information

**Additional file 1**. **Tab. S1.** Antibody panels used to identify immune cells in blood and broncho-alveolar lavage fluid. **Fig. S1.** The effect of housing condition on growth performance, T helper cells and memory T helper cells in blood, as well as memory Thelper cells in broncho-alveolar lavage fluid. **Fig. S2.** The effect of time on faecal microbial beta-diversity and alphadiversity. **Fig. S3.** The effect of gut location on luminal microbial beta-diversity and alpha-diversity. **Fig. S4.** The effect of housing condition on faecal microbial variation on day 12 and day 26. **Fig. S5.** The effect of housing condition on faecal microbial alpha-diversity over time. **Fig. S6.** The genera that were differentially abundant between both housing conditions. **Fig. S7.** Distance-based redundancy analysis (db-RDA) triplot showing the association between fecal microbiota variation and environmental variables based on weighted uniFrac distance. **Fig. S8.** Linear Discriminant Analysis (LDA) Effect Size (LEfSe) plot of differentially abundant ileal taxa between CH (conventional housing) and EH (enriched housing) pigs on day 61.

## Data Availability

The sequence data was submitted to European Nucleotide Archive with accession number PRJEB44226.

## Abbreviations

NK cells: Natural Killer Cells
IL-1ß: Interleukin 1 beta
IL-6: Interleukin61
TNF-α: Tumor necrosis factor alpha
TLR4: Toll-like receptor 4
CD: Cluster of differentiation
GLIMMIX: Generalized linear mixed model
