# Supplementary material for: Environmentally enriched housing conditions affect pig welfare, immune system and gut microbiota in early life
Source: Anim Microbiome. 2021 Jul 28;3:52. doi: 10.1186/s42523-021-00115-2 (PMC8320228; doi:10.1186/s42523-021-00115-2)
Supplement: Supplementary file 1 — Additional file 1. Tab. S1. Antibody panels used to identify immune cells in blood and broncho-alveolar lavage fluid. Fig. S1. The effect of housing condition on growth performance, T helper cells and memory T helper cells in blood, as well as memory Thelper cells in broncho-alveolar lavage fluid. Fig. S2. The effect of time on faecal microbial beta-diversity and alphadiversity. Fig. S3. The effect of gut location on luminal microbial beta-diversity and alpha-diversity. Fig. S4. The effect of housing condition on faecal microbial variation on day 12 and day 26. Fig. S5. The effect of housing condition on faecal microbial alpha-diversity over time. Fig. S6. The genera that were differentially abundant between both housing conditions. Fig. S7. Distance-based redundancy analysis (db-RDA) triplot showing the association between fecal microbiota variation and environmental variables based on weighted uniFrac distance. Fig. S8. Linear Discriminant Analysis (LDA) Effect Size (LEfSe) plot of differentially abundant ileal taxa between CH (conventional housing) and EH (enriched housing) pigs on day 61. [file 42523_2021_115_MOESM1_ESM.docx]

**Tab. S1** **Antibody panels used to identify immune cells in blood and broncho-alveolar lavage fluid.**

|  |  | Antigen | Clone | Isotype | Fluorochrome | Labelling | Source of antibody |
| --- | --- | --- | --- | --- | --- | --- | --- |
| Blood cells | Single staining | CD172a | 74-22-15 | IgG2b | FITC | Secondary^1^ | VMRD |
|  |  | CD21 | B6-11C9 | IgG1 | APC | Secondary^1^ | Southern Biotech |
|  | Triple staining | CD3 | PPT3 | IgG1 | APC | Secondary^1^ | Southern Biotech |
|  |  | CD4 | 74-12-4 | IgG2b | FITC | Secondary^1^ | Southern Biotech |
|  |  | CD8α | 76-2-11 | IgG2a | PE | Secondary^1^ | Southern Biotech |
| BALF cells | Single staining | Granulocytes | 2B2 | IgG1 | FITC |  | Serotec |
|  | Double staining | CD172a | 74-22-15 | IgG2b | PE | Secondary^1^ | VMRD |
|  |  | CD172a | 74-22-15 | IgG1 | PE | Secondary^1^ | Beckman Coulter |
|  |  | CD 14 | MIL2 | IgG2b | FITC | Primary | BioSource |
|  |  | TLR4 | clone 11E10 | IgM | FITC | Secondary^1^ | Gift by J. Dominguez |
|  | Triple staining | CD3 | PPT3 | gG1 | APC | Secondary^1^ | Southern Biotech |
|  |  | CD4 | 74-12-4 | IgG2b | FITC | Secondary^1^ | Southern Biotech |
|  |  | CD8α | 76-2-11 | IgG2a | PE | Secondary^1^ | Southern Biotech |

**
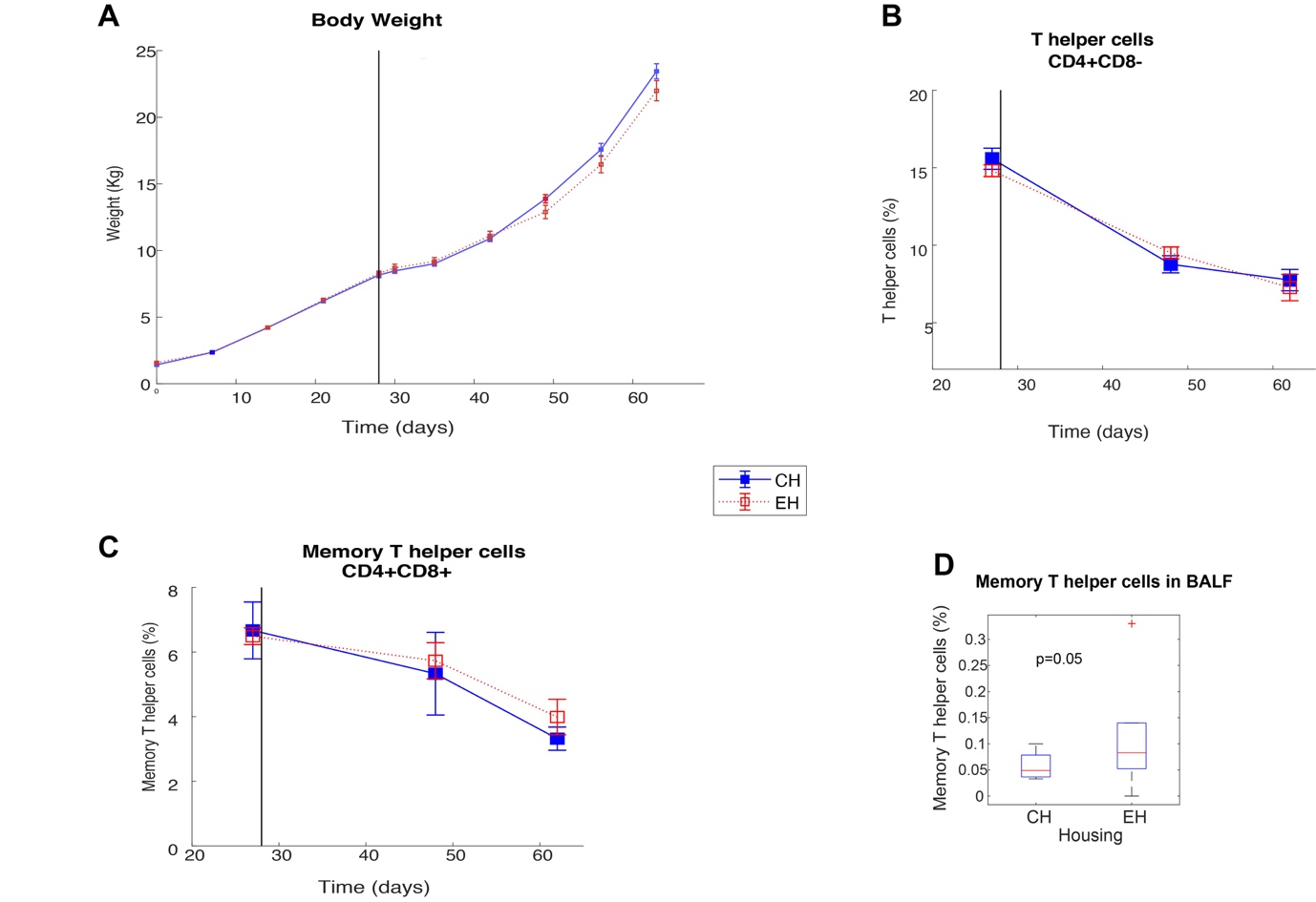
**

**Fig. S1. The effect of housing condition on growth performance, T helper cells and memory T helper cells in blood, as well as memory T helper cells in broncho-alveolar lavage fluid.** The housing condition did not affect piglets growth performance (**A**), T helper cells and memory T helper cells in blood (**B&C**). Conventional housing (CH) and enriched housing (EH) are represented by colour blue and red, respectively. Housing environment influcenced the percentage of memory T helper cells in broncho-alveolar lavage fluid (BALF) on day 61 (**D**).


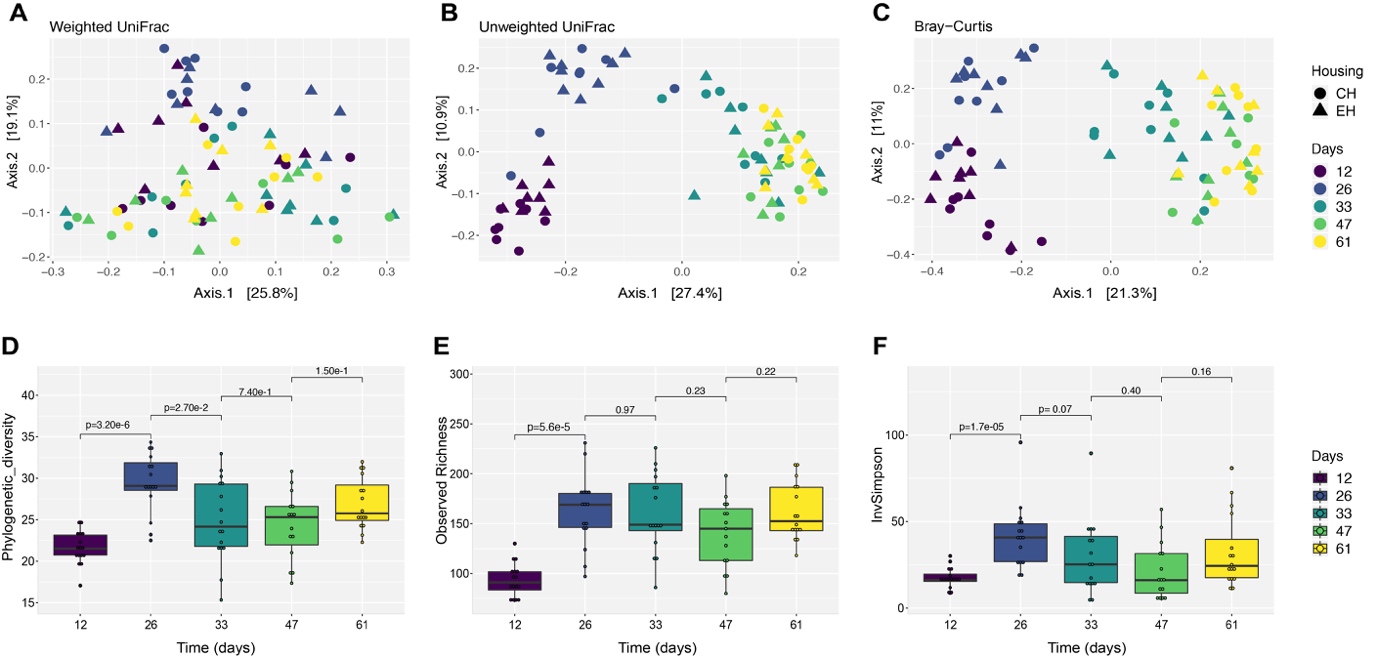


**Fig. S2** **The effect of time on faecal microbial beta-diversity and alpha-diversity**. Principal Coordinates Analysis (PCoA) plot of beta-diversity based on weighted uniFrac (**A**), unweighted uniFrac (**B**) distance and Bray-Curtis dissimilarity (**C)** in faecal samples over time (day 12-61). Conventional housing (CH) and enriched housing (EH) are represented by circles () and triangles (□), respectively. Boxplots show alpha-diversity based on phylogenetic_diversity (**D**), observed richness (**E**) and InSimpson (**F**) in fecal samples over time (day 12-61). Differences between timepoints was evaluated by nonparametric Wilcoxon rank sum test.


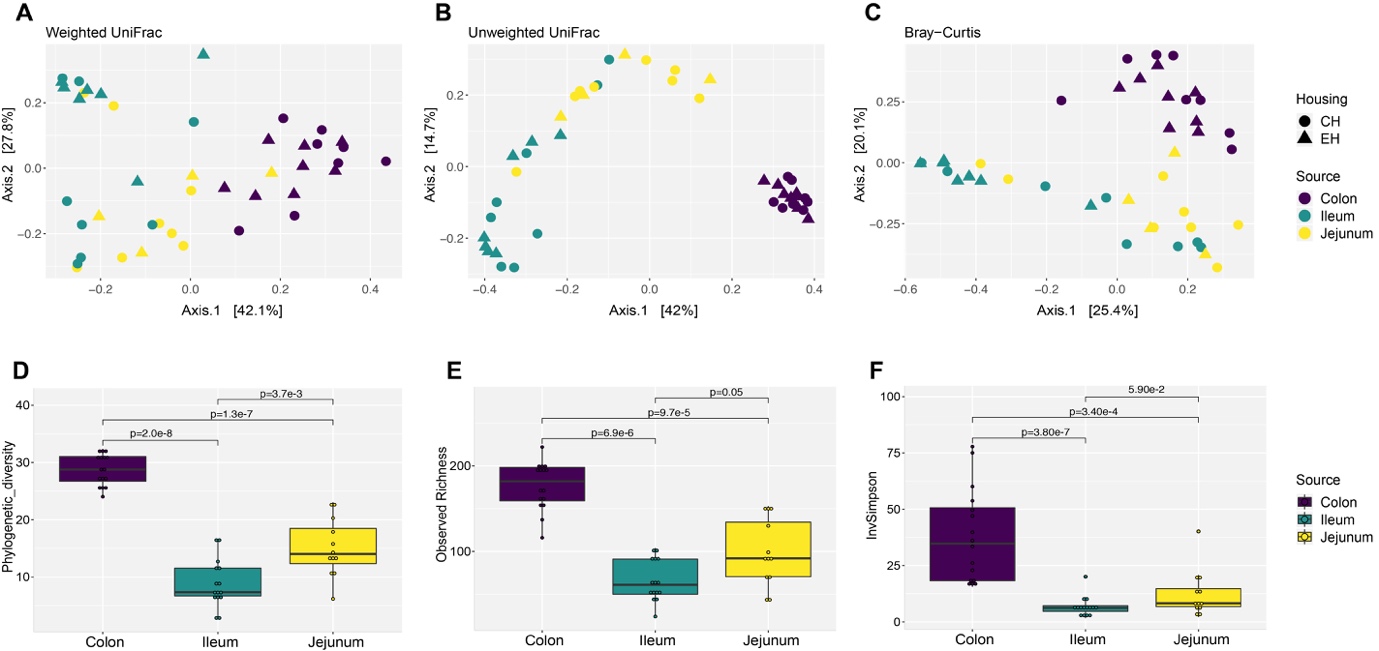


**Fig. S3** **The effect of gut location on luminal microbial beta-diversity and alpha-diversity**. Principal Coordinates Analysis (PCoA) plot of beta-diversity based on weighted uniFrac (**A**), unweighted uniFrac (**B**) distance and Bray-Curtis dissimilarity (**C**) in luminal digesta samples. Conventional housing (CH) and enriched housing (EH) are represented by circles () and triangles (□), respectively. Alpha-diversity was indicated based on phylogenetic_diversity (**D**), observed richness (**E**) and InSimpson (**F**) and differences between each location was evaluated by nonparametric Wilcoxon rank sum test.


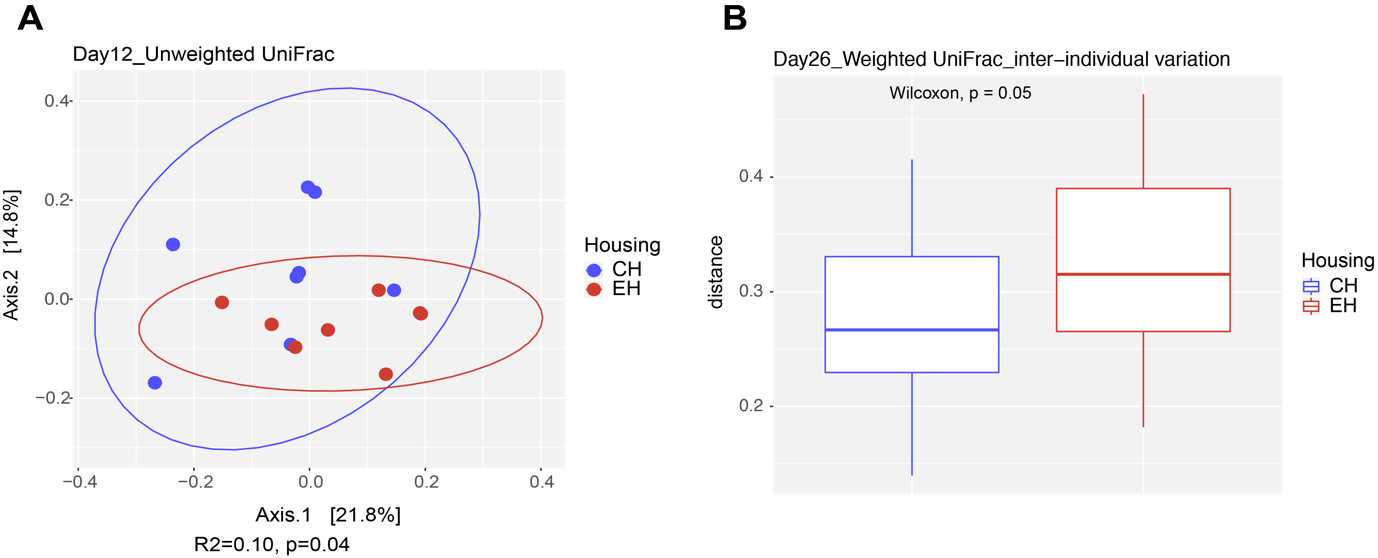


**Fig. S4** **The effect of housing condition on faecal microbial variation on day 12 and day 26.** Principal coordinate analysis (PCoA) plots based on unweighted UniFrac matrix (**A**) using data from day 12 and significance of the difference between conventional housing (CH) and enriched housing (EH) was assessed using PERMANOVA. Differences in inter-individual variation for CH versus EH pigs in faecal microbiota composition on day 26 as assessed by Wilcoxon rank sum test (**B**).


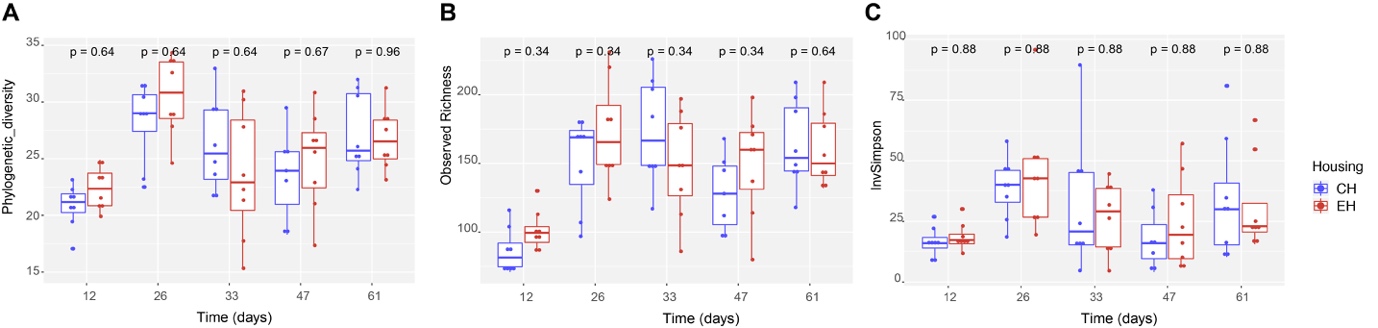


**Fig. S5** **The effect of housing condition on faecal microbial alpha-diversity over time.** Comparisons of alpha-diversity between both housing conditions in fecal samples at each timepoint was assessed based on phylogenetic_diversity (**A**), observed richness (**B**) and InSimpson (**C**). Differences between each location was evaluted by nonparametric Wilcoxon rank sum test.


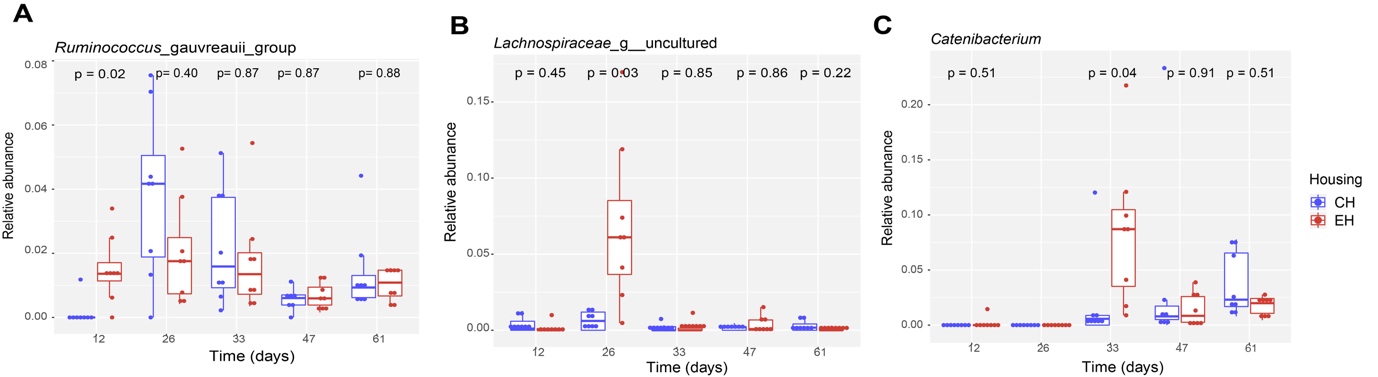


**Fig. S6 The genera that were differentially abundant between both housing conditions.** Box plots showing three genera that were significantly different between both housing conditions at least on one of the sampling days, (**A**) Ruminococcus_gauvreauii_group on day 13, (**B**) Lachnospiraceae_g_uncultured on day 26 and Cantenibacterium (**C**) on day 33. Differences were evaluted by nonparametric Wilcoxon rank sum test. Blue and red colours represent conventional housing (CH) and enriched housing (EH), respectively.


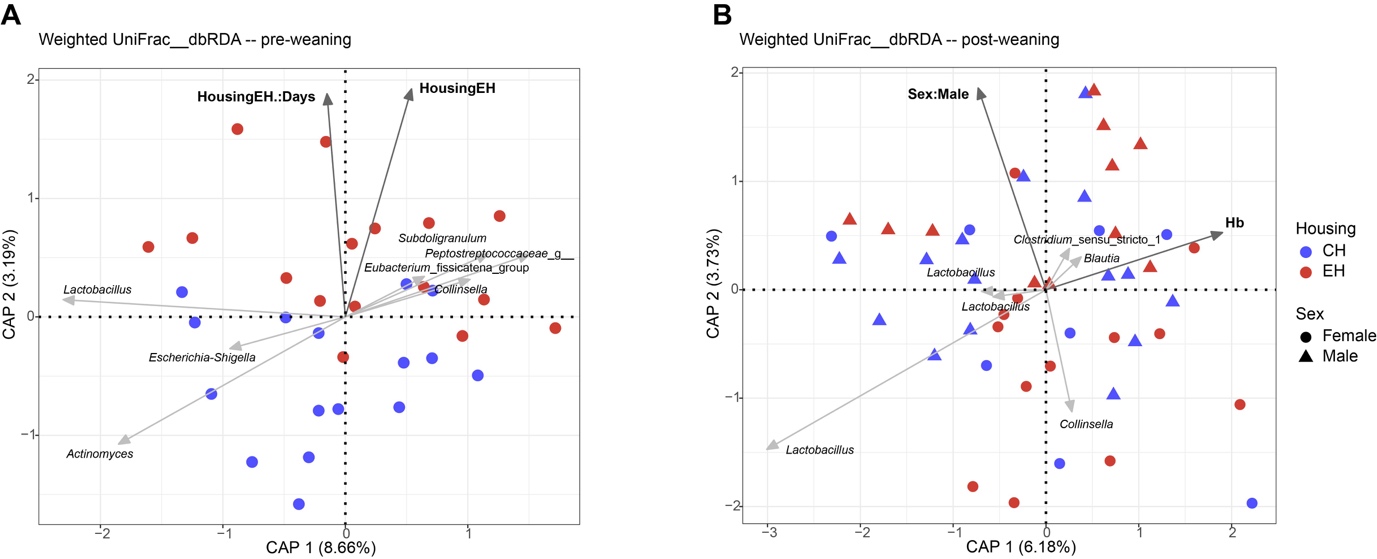


**Fig. S7** **Distance-based redundancy analysis (db-RDA) triplot showing the association between fecal microbiota variation and environmental variables based on weighted uniFrac distance**. (**A**) focusing on samples from pre-weaning (day 12 and day 26), (**B**) focusing on samples from post-weaning (day 33, day 47 and day 61). Colours represent different housing conditions (conventional housing: CH; enriched housing: EH) and shapes show different sex. Dark grey arrows indicate environmental variables and light grey arrows ASVs for which the model provided the best fit for the observed variation. The factor time (Days) was taken as the conditional variable in db-RDA analysis.

**
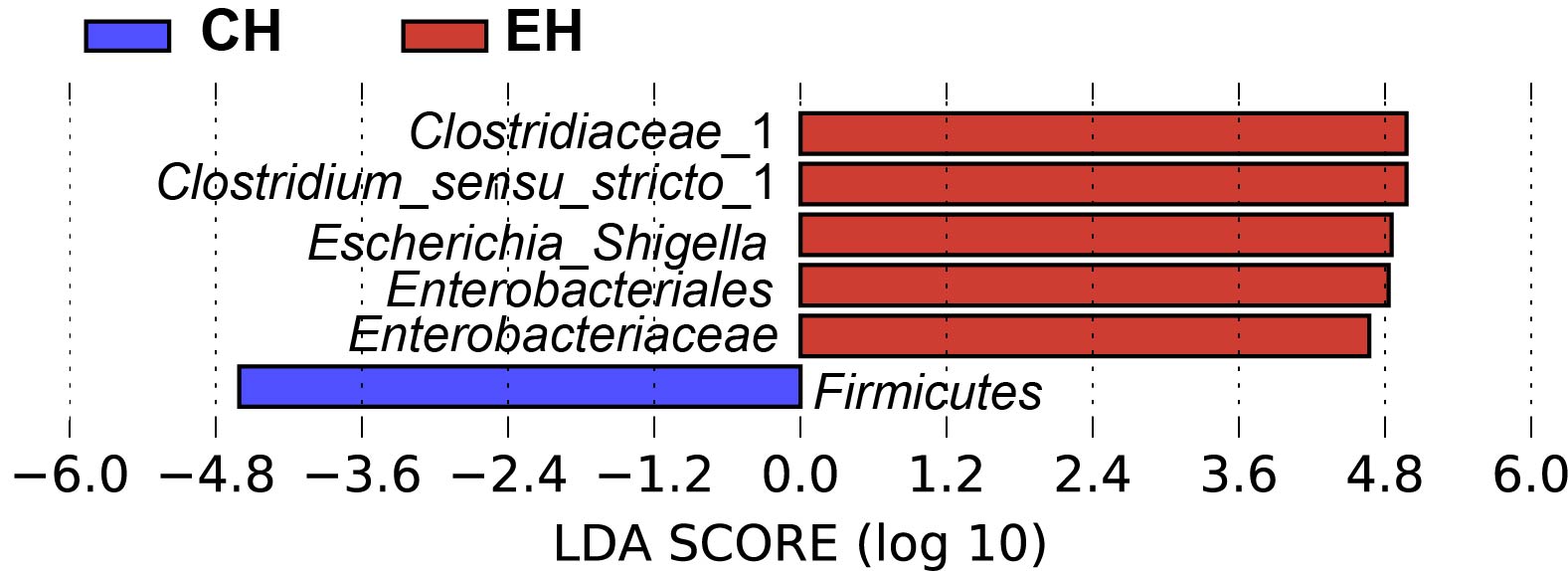
**

**Fig. S8** **Linear Discriminant Analysis (LDA) Effect Size (LEfSe) plot of differentially abundant ileal taxa between CH (conventional housing) and EH (enriched housing) pigs on day61.**
